# The impact of *FLT3* mutation clearance and treatment response after gilteritinib therapy on overall survival in patients with *FLT3* mutation–positive relapsed/refractory acute myeloid leukemia

**DOI:** 10.1002/cam4.3652

**Published:** 2020-12-19

**Authors:** Jessica K. Altman, Alexander E. Perl, Jason E. Hill, Matt Rosales, Erkut Bahceci, Mark J. Levis

**Affiliations:** ^1^ Robert H Lurie Comprehensive Cancer Center Northwestern University Chicago IL USA; ^2^ Abramson Comprehensive Cancer Center University of Pennsylvania Philadelphia PA USA; ^3^ Astellas Pharma US, Inc. Northbrook IL USA; ^4^ Sidney Kimmel Comprehensive Cancer Center Johns Hopkins University Baltimore MD USA

**Keywords:** FLT3 inhibitor, *Fms*‐like tyrosine kinase 3, internal tandem duplication, morphologic remission

## Abstract

The FLT3 inhibitor gilteritinib has clinical activity in patients with *FLT3*‐mutated (*FLT3* ^mut+^) relapsed/refractory (R/R) acute myeloid leukemia (AML). The impact of *FLT3* mutation clearance and the achievement of composite complete remission (CRc) and complete remission/complete remission with partial hematologic recovery (CR/CRh) on overall survival (OS) in patients with *FLT3* ^mut+^ R/R AML treated with single‐agent gilteritinib in a phase 1/2 trial were evaluated. Using next‐generation sequencing, a *FLT3*‐ITD variant allele frequency of ≤10^−4^ was used to define *FLT3*‐ITD clearance in patients with no morphologic leukemia (ie, CRc). A total of 108 patients with *FLT3*‐ITD‐positive (*FLT3*‐ITD+) R/R AML were analyzed; 95 of these patients had received ≥80‐mg/day gilteritinib. Ten of the 95 patients had *FLT3*‐ITD clearance; eight of these 10 patients achieved CRc and were considered negative for measurable residual disease. There was a trend toward longer OS in patients who attained CRc with *FLT3*‐ITD clearance (131.4 weeks) versus those who achieved CRc and did not have *FLT3*‐ITD clearance (*n* = 41; 43.3 weeks; HR = 0.416; *p* = 0.066). Among patients treated with ≥80‐mg/day gilteritinib who achieved CR/CRh (*n* = 24), seven had *FLT3*‐ITD clearance. Among patients who received 120‐mg/day gilteritinib, those who achieved CR/CRh had a longer median OS (70.6 weeks) and higher 52‐week survival probability (66.7%) than patients who did not achieve CR/CRh (*n* = 71; median OS, 41.7 weeks; 52‐week survival probability, 20.2%). Overall, these data suggest that gilteritinib can induce deep molecular responses in patients with *FLT3*‐ITD+ R/R AML, and in the setting of CRc or CR/CRh, these responses may be associated with prolonged survival.

## Graphical abstract

In patients with *FLT3*‐mutated relapsed/refractory acute myeloid leukemia, the *FLT3* inhibitor gilteritinib induced deep molecular responses characterized by *FLT3*–internal tandem duplication (*FLT3*‐ITD) mutation clearance. Among patients who achieved morphologic remission with gilteritinib therapy, *FLT3*‐ITD mutation clearance was associated with a trend toward longer survival.

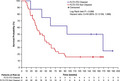

## 1 INTRODUCTION

Measurable residual disease (MRD) in AML refers to the persistence of residual leukemic cells below clinically detectable levels despite achievement of morphologic remission.^1^ The presence of MRD after achievement of morphologic complete remission (CR) has emerged as an independent prognostic marker of increased relapse risk and shortened survival in patients with AML.^1^, ^2^

Common techniques to detect MRD include multi‐parameter flow cytometry (MFC) and reverse transcriptase quantitative polymerase chain reaction (RT‐qPCR). Despite the wide applicability of MFC, the sensitivity of detection of leukemia‐associated immunophenotypic cells is only 0.1% to 0.01% (10^−3^ to 10^−4^) and requires comparison against diagnostic specimens, which are not always available.^3^ Although use of optimized RT‐qPCR to detect MRD by amplifying leukemia‐associated genetic aberrations has increased sensitivity to a 10^−4^ to 10^−6^ range, these assays depend on the presence of specific mutations (*NPM1* and *core*‐*binding factor*) and require standard curves for reference.^1^ Digital PCR improves on RT‐qPCR sensitivity without the need for a standard curve.^1^ Next‐generation sequencing (NGS) has emerged as a valuable tool for monitoring MRD in AML because it enables comprehensive and simultaneous detection of patient‐specific somatic mutations indicating subclinical disease that is undetected by RT‐qPCR, has a sensitivity of 10^−3^ to 10^−6^, and is applicable in the majority of AML cases.^2^, ^4^, ^5^

In AML, the likelihood of MRD after chemotherapy is substantially greater in patients harboring activating *FLT3* internal tandem duplication (*FLT3*‐ITD) mutations than in patients with wild‐type *FLT3*.^6^ However, as *FLT3*‐ITD mutations do not always persist at relapse, systematic monitoring of *FLT3*‐ITD mutations in patients in first remission remains controversial.^7^ With the emergence of approved FLT3 inhibitors for AML, accurate assessment of MRD will establish the long‐term efficacy of these agents. Conventional PCR assays using genomic DNA for *FLT3* mutation detection are confounded by template bias from the wild‐type allele,^8^ resulting in low sensitivity (~1%). This challenge can be overcome through a combined PCR‐NGS approach, with bioinformatics designed to detect *FLT3*‐ITD insertions of normal coding sequence. This approach enables identification of clonal composition and the dominance of *FLT3*‐ITD mutations.^9^, ^10^ Although *FLT3*‐ITD–positive (+) clones are unstable, appearing or disappearing at relapse,^11^ considerable inter‐individual heterogeneity in ITD length^12^, ^13^ facilitates detection specificity.^9^ A clinical response to a FLT3 inhibitor without a reduction in *FLT3* allele burden may reflect differentiation of the leukemic clone.^14^, ^15^

Gilteritinib is an oral FLT3 inhibitor with activity against *FLT3*‐ITD and *FLT3* tyrosine kinase domain (TKD) mutations.^16^, ^17^ In the phase 1/2 CHRYSALIS study, gilteritinib was well tolerated and induced antileukemic activity at doses ≥80 mg/day in patients with relapsed/refractory (R/R) *FLT3*‐mutated (*FLT3* ^mut+^) AML.^18^ The phase 3 ADMIRAL trial demonstrated the superior efficacy of 120‐mg/day gilteritinib compared with salvage chemotherapy in patients with R/R *FLT3* ^mut+^ AML.^19^, ^20^

A sensitive and specific NGS‐based assay used to detect MRD in a *FLT3*‐ITD+ R/R AML patient subset who achieved morphologic CR with gilteritinib in the CHRYSALIS study demonstrated a relationship between *FLT3*‐ITD mutation burden and overall survival (OS).^9^

FLT3 inhibitors can induce differentiation and cytotoxicity in bone marrow blasts, resulting in CR with incomplete hematologic recovery (CRi).^18^, ^21^ Studies of FLT3 inhibitors in R/R AML report the composite CR (CRc) rate, which is the sum of CR, CRi, and CR with incomplete platelet recovery (CRp).^18^, ^21^ A CRh response describes bone marrow blast clearance with partial, clinically significant, hematologic recovery that is not defined by other response criteria.^22^ The impact of CRc or CR/CRh on *FLT3*‐ITD clearance and long‐term survival in patients with *FLT3* ^mut+^ R/R AML has not been evaluated.

A previous analysis of a *FLT3*‐ITD+ R/R AML patient subgroup from the CHRYSALIS study who achieved CRc with 120‐mg or 200‐mg gilteritinib demonstrated longer survival in MRD‐negative (MRD−) patients than in MRD‐positive (MRD+) patients over a 1‐ to 2‐year period.^9^ We evaluated the impact of *FLT3*‐ITD mutation clearance and achievement of CRc or CR/CRh on survival beyond 3 years in a larger *FLT3*‐ITD+ R/R AML patient subset who received 20‐ to 450‐mg/day gilteritinib in the CHRYSALIS study.^18^

## 2 METHODS

### 2.1 CHRYSALIS study design and patient population

CHRYSALIS was a phase 1/2, open‐label, dose‐escalation/dose‐expansion study of once‐daily oral gilteritinib (20–450 mg) in patients with R/R AML.^18^ Adult patients (aged ≥18 years) with primary or secondary AML who were either refractory to ≥1 cycle of induction chemotherapy, or had relapsed after achieving remission with a prior therapy, were enrolled.^18^ Patients with *FLT3*‐ITD mutations or *FLT3*‐TKD point mutations were enrolled into each expanded dose level, including the dose‐escalation cohorts (20‐, 40‐, 80‐, 120‐, 200‐, 300‐, and 450‐mg/day gilteritinib).^18^ The decision to escalate to the next dose level was based on the assessment of grade 2 adverse events and dose‐limiting toxicities.^18^

### 2.2 Assessment of mutation clearance

The presence of *FLT3*‐ITD mutations was assessed in bone marrow aspirates from patients with *FLT3*‐ITD+ R/R AML who had received 20‐ to 450‐mg/day gilteritinib and had samples available at baseline and ≥1 additional post‐baseline time point prior to hematopoietic stem cell transplantation (HSCT). The *FLT3*‐ITD mutation assay was performed according to previously published methods.^9^ Specifically, using genomic DNA, *FLT3* exons 14 and 15 were amplified by PCR and *FLT3*‐ITD and total *FLT3* alleles were subsequently quantified by NGS using an Illumina^®^ MiSeq platform.^9^ Read depths of ≥100,000 reads per sample were implemented, and operating characteristics were linear to 10^−4^ for the range of ITD lengths using cell lines spiked to normal blood or bone marrow.^9^ Data were analyzed using proprietary software. *FLT*3‐ITD variant allele frequency (VAF) was defined as the *FLT3*‐ITD to total *FLT3* frequency; *FLT3*‐ITD clearance was defined as a *FLT3*‐ITD VAF of ≤10^−4^. A patient was classified as having *FLT3*‐ITD clearance if they had a *FLT3*‐ITD VAF of ≤10^−4^ at any post‐baseline time point prior to HSCT. A Cox regression model, with Kaplan–Meier estimation, was used to evaluate the impact of *FLT3* VAF on OS.

### 2.3 Assessment of the relationship between treatment response, mutation clearance, and OS

The impact of CRc or CR/CRh with *FLT3*‐ITD mutation clearance on OS was assessed in a subgroup of patients with *FLT3*‐ITD+ R/R AML who had received ≥80‐mg/day gilteritinib, which induced maximum inhibition of FLT3 receptor auto‐phosphorylation and antileukemic response.^18^ The impact of achieving CR/CRh on OS was assessed in a subgroup of patients with *FLT3*‐ITD+ R/R AML who had received 120 mg/day, which was established as the recommended dose in the phase 1/2 CHRYSALIS study,^18^ and was evaluated against salvage chemotherapy in the phase 3 ADMIRAL trial.^20^ Composite complete remission was defined as CR plus CRp plus CRi. Patients who achieved CR achieved morphologic leukemia‐free status, had an absolute neutrophil count (ANC) of >1 × 10^9^/L and an absolute platelet count of ≥100 × 10^9^/L, normal bone marrow differential with <5% bone marrow blasts, red blood cell and platelet transfusion independence, and no evidence of extramedullary leukemia. Patients who achieved CRp met all of the criteria for CR but had a platelet count of <100 × 10^9^/L in the absence of transfusions. Patients who achieved CRi met all of the criteria for CR but had residual neutropenia (ANC < 1 × 10^9^/L) with or without platelet recovery. Patients who achieved CRh had bone marrow blasts <5%, ANC ≥ 0.5 × 10^9^/L, absolute platelet count of ≥50 × 10^9^/L, and no evidence of extramedullary leukemia.

### 2.4 Statement of ethics

The CHRYSALIS study was conducted in accordance with the ethical principles of the Declaration of Helsinki, Good Clinical Practice guidelines, the principles of informed consent, and the requirements of public registration of clinical trials. Approval was obtained from site‐specific institutional review boards. Written informed consent was obtained from each patient at the time of enrollment.

## 3 RESULTS

### 3.1 Analysis population

A total of 108 patients out of 178 with *FLT3*‐ITD+ R/R AML in the CHRYSALIS study were analyzed for *FLT3*‐ITD mutation clearance. Of these patients, 40 (37%) had samples available for analysis at one or two post‐baseline time points (treatment cycles 3 and 4) and 21 (19.4%) had samples available for analysis at >2 post‐baseline time points (up to treatment cycle 28). Patients with evidence of *FLT3*‐ITD mutation clearance only after HSCT were classified as not having *FLT3*‐ITD clearance.

Demographic and baseline characteristics of patients assessed for mutation clearance were representative of the entire CHRYSALIS R/R AML population (Table 1).^18^ Overall, 95 of 108 patients (88%) had received ≥80‐mg/day gilteritinib. *NPM1* mutations (based on local laboratory testing) were also present in 37 of 108 patients (34.3%)—33 of whom had received ≥80‐mg/day gilteritinib. A total of 10 of the 108 patients had achieved *FLT3*‐ITD mutation clearance with gilteritinib therapy; all 10 of these patients had received ≥80‐mg doses of gilteritinib. Median *FLT3*‐ITD VAF at baseline was similar in patients with *FLT3*‐ITD mutation clearance and in those without *FLT3*‐ITD mutation clearance; median ITD length was 48 bp (range, 3–204). Among all patients receiving gilteritinib (*n* = 108), CR rates with prior AML therapy were higher among patients with *FLT3*‐ITD mutation clearance (80%; *n* = 8/10) than in those without *FLT3*‐ITD mutation clearance (58%; *n* = 57/98), and the median duration of CR achieved with prior AML therapy was also longer in patients with *FLT3*‐ITD mutation clearance (7.9 vs. 4.1 months, respectively).

**Table 1:** Demographic and baseline characteristics of patients with *FLT3*‐ITD+ R/R AML who were assessed for *FLT3* mutation clearance

Characteristics	Gilteritinib ≥ 80 mg/day subgroup			Total analysis population		
	*FLT3*‐ITD Cleared (*n* = 10)	*FLT3*‐ITD Not Cleared (*n* = 85)	Combined (*N* = 95)	*FLT3*‐ITD Cleared (*n* = 10)	*FLT3*‐ITD Not Cleared (*n* = 98)	Combined (*N* = 108)
Median age, years (range)	59.5 (29–76)	61.0 (21–86)	61.0 (21–86)	59.5 (29–76)	60.5 (21–86)	60.5 (21–86)
Median *FLT3* variant allele frequency (range)	0.4205 (0.041–0.827)	0.407 (0.036–0.986)	0.407 (0.036–0.986)	0.4205 (0.041–0.827)	0.4056 (0.036–0.986)	0.4056 (0.036–0.986)
Sex, *n* (%)						
Male	3 (30)	42 (49)	45 (48)	3 (30)	49 (50)	52 (48)
Female	7 (70)	43 (51)	50 (53)	8 (70)	49 (50)	56 (52)
AML type, *n* (%)						
De novo	9 (90)	72 (85)	81 (85)	9 (90)	83 (85)	92 (85)
Secondary	1 (10)	13 (15)	14 (15)	1 (10)	15 (15)	16 (15)
Cytogenetic risk status, *n* (%)						
Favorable	1 (10)	2 (2)	3 (3)	1 (10)	2 (2)	3 (3)
Intermediate	8 (80)	56 (66)	64 (67)	8 (80)	65 (66)	73 (68)
Unfavorable	0	12 (14)	12 (13)	0	14 (14)	14 (13)
Unknown/missing	1 (10)	15 (18)	16 (17)	1 (10)	17 (17)	18 (17)
Lines of prior AML therapy, *n* (%)						
1	4 (40)	29 (34)	33 (35)	4 (40)	32 (33)	36 (33)
2	3 (30)	25 (29)	28 (29)	3 (30)	27 (28)	30 (28)
≥3	3 (30)	31 (36)	34 (36)	3 (30)	39 (40)	42 (39)
Prior transplantation, *n* (%)						
Yes	5 (50)	20 (24)	25 (26)	5 (50)	22 (22)	27 (25)
No	5 (50)	65 (76)	70 (74)	5 (50)	76 (78)	81 (75)
Prior TKI therapy, *n* (%)						
Yes	3 (30)	24 (28)	27 (28)	3 (30)	30 (31)	33 (31)
No	7 (70)	61 (72)	68 (72)	7 (70)	68 (69)	75 (69)
Best response to prior AML therapy, *n* (%)						
CR	8 (80)	49 (58)	57 (60)	8 (80)	57 (58)	65 (60)
PR	1 (10)	16 (19)	17 (18)	1 (10)	17 (17)	18 (17)
Duration of response to prior AML therapy						
Median duration of CR, months (range)	7.9 (1.7–15.8)	3.9 (0.8–64.1)	4.6 (0.8–64.1)	7.9 (1.7–15.8)	4.1 (0.8–64.1)	4.9 (0.8–64.1)
Median duration of PR, months (range)	1.0 (1.0–8.9)	1.3 (0.4–12.0)	1.0 (0.4–12.0)	1.0 (1.0–8.9)	1.7 (0.4–12.0)	1.3 (0.4–12.0)

Percentages are rounded to whole numbers.

Abbreviations: AML, acute myeloid leukemia; CR, complete remission; ITD, internal tandem duplication; PR, partial remission; R/R, relapsed/refractory; TKI, tyrosine kinase inhibitor.

### 3.2 Overall survival according to mutation clearance status

Among the 95 patients who received ≥80‐mg/day gilteritinib, 10 had *FLT3*‐ITD clearance at any post‐baseline time point. Median OS was 76.8 weeks (95% CI: 18.6, not reached) in patients who had *FLT3*‐ITD clearance and 30.6 weeks (95% CI: 22.4, 37.7) in those who did not have *FLT3*‐ITD clearance (HR = 0.663; 95% CI: 0.298, 1.475).

### 3.3 Impact of mutation clearance and achievement of CRc on overall survival

**Figure 1:**
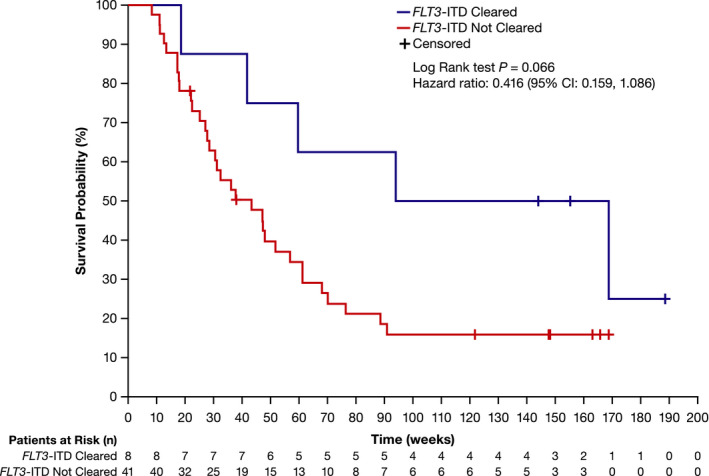
Overall survival in patients with *FLT3*‐ITD+ R/R AML who received ≥80‐mg/day gilteritinib and had a best overall response of CRc stratified by *FLT3* mutation clearance status. AML, acute myeloid leukemia; CI, confidence interval; CRc, composite complete remission; ITD, internal tandem duplication; and R/R, relapsed/refractory

Of the 95 patients who received ≥80‐mg/day gilteritinib, 49 (51.6%) had a best overall response of CRc (Table  2). Patients who achieved CRc and had *FLT3*‐ITD clearance (*n* = 8) had a trend toward longer median OS (131.4 weeks; 95% CI: 18.6, not reached) than those who achieved CRc and did not have *FLT3*‐ITD clearance (*n* = 41; median OS: 43.3 weeks; 95% CI: 27.7, 56.9; HR = 0.416 [95% CI: 0.159, 1.086]) and did not reach statistical significance (*p* = 0.066; Figure 1). Two of the eight patients (25.0%) who achieved CRc with *FLT3*‐ITD clearance underwent HSCT after achieving CRc. Of the 41 patients who achieved CRc without *FLT3*‐ITD clearance, 15 (36.6%) underwent HSCT after achieving CRc.

**Table 2:** *FLT3* mutation clearance in patients with *FLT3*‐ITD R/R AML according to CRc response after treatment with ≥80‐mg/day gilteritinib

Mutation clearance status	CRc (*n* = 49)	No CRc (*n* = 46)	Total (*N* = 95)
*FLT3*‐ITD cleared	8 (16.3%)	2 (4.3%)	10 (10.5%)
*FLT3*‐ITD not cleared	41 (83.7%)	44 (95.7%)	85 (89.5%)

Abbreviations: AML, acute myeloid leukemia; CRc, composite complete remission; ITD, internal tandem duplication; R/R, relapsed/refractory.

**Figure 2:**
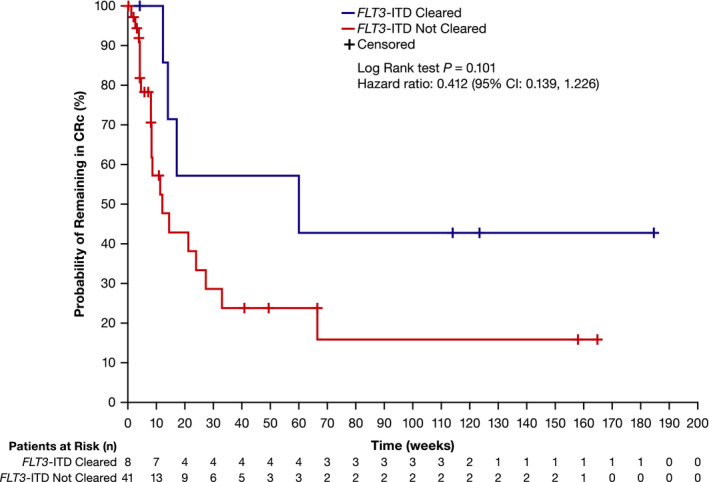
Duration of CRc according to *FLT3* mutation clearance status in patients with *FLT3*‐ITD+ R/R AML who received ≥80‐mg/day gilteritinib. AML, acute myeloid leukemia; CI, confidence interval; CRc, composite complete remission; ITD, internal tandem duplication; MRD, minimal residual disease; and R/R, relapsed/refractory

Overall, the median duration of CRc in patients who had *FLT3*‐ITD clearance was 60.0 weeks (95% CI: 12.3, not reached) and 12.1 weeks (95% CI: 8.3–27.3) in those who did not have *FLT3*‐ITD clearance (HR = 0.412; 95% CI: 0.139, 1.226; Figure 2). Among patients who had received ≥80‐mg/day gilteritinib and had prior treatment with a FLT3 TKI (*n* = 27), two of three patients achieved CRc with *FLT3*‐ITD clearance; 11 of 24 patients achieved CRc without *FLT3*‐ITD clearance.

### 3.4 Impact of *FLT3* mutation clearance status and achievement of CR/CRh on OS

**Figure 3:**
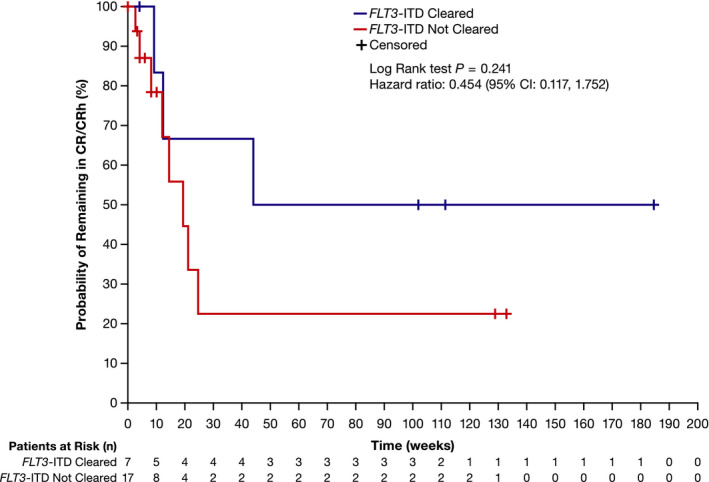
Duration of CR/CRh according to *FLT3* mutation clearance status in patients with *FLT3*‐ITD+ R/R AML who received ≥80‐mg/day gilteritinib. AML, acute myeloid leukemia; CI, confidence interval; CR, complete remission; CRh, complete remission with partial hematologic recovery; ITD, internal tandem duplication; and R/R, relapsed/refractory

Overall, 24 of 95 patients (25.3%) who received ≥80‐mg/day gilteritinib had a best overall response of CR/CRh; 71 patients did not achieve CR/CRh (Table 3). Of the 24 patients with CR/CRh, seven (29.2%) had *FLT3*‐ITD clearance and 17 (70.8%) did not have *FLT3*‐ITD clearance. Of the seven patients with *FLT3*‐ITD clearance, five (71.4%) achieved CR and two (28.6%) achieved CRh. Of the 17 who did not have *FLT3*‐ITD clearance, seven (41.2%) achieved CR and 10 (58.8%) achieved CRh. Three of the 71 patients who did not achieve CR/CRh (4.2%) had *FLT3*‐ITD clearance. For patients who achieved CR/CRh and had *FLT3*‐ITD clearance, the median duration of CR/CRh had not been reached; those who did not have *FLT3*‐ITD clearance had a median duration of CR/CRh of 19.4 weeks (HR = 0.454; 95% CI: 0.117, 1.752; Figure 3).

**Table 3:** *FLT3* mutation clearance in patients with *FLT3*‐ITD+ R/R AML according to CR/CRh response after treatment with ≥80‐mg/day gilteritinib

Mutation clearance status	CR/CRh (*n* = 24)	No CR/CRh (*n* = 71)	Total (*N* = 95)
*FLT3*‐ITD cleared	7 (29.2%)	3 (4.2%)	10 (10.5%)
*FLT3*‐ITD not cleared	17 (70.8%)	68 (95.8%)	85 (89.5%)

Abbreviations: AML, acute myeloid leukemia; CR, complete remission; CRh, complete remission with partial hematologic recovery; ITD, internal tandem duplication; R/R, relapsed/refractory.

Based on aggregate data pertaining to the toxicity and antileukemic activity of gilteritinib, a dose of 120 mg/day was selected as the starting dose of gilteritinib as single‐agent therapy in subsequent clinical trials. Of the 56 *FLT3*‐ITD+ patients in the 120‐mg/day dose cohort, 34 were assessed for *FLT3* mutation clearance, and six of the 34 patients (17.6%) had *FLT3*‐ITD clearance. Nine of the 34 patients (26.5%) achieved a best overall response of CR/CRh. After excluding patients with an OS duration that was less than the median time to reach CR/CRh, patients who achieved CR/CRh had a longer median OS and greater survival probability than patients who did not achieve CR/CRh. The median OS in the CR/CRh and no CR/CRh cohorts was 70.6 weeks (95% CI: 27.1, not reached) and 41.7 weeks (95% CI: 30.4, 51.7), respectively (Figure S1). The survival probability at 52 weeks was 66.7% (95% CI: 33.7, 86.0) versus 20.2% (95% CI: 9.5, 33.6), respectively.

## 4 DISCUSSION

Expanding knowledge of the genomic landscape in AML, as well as the recent availability of multiple targeted therapies for patients with newly diagnosed or R/R AML, has substantially reshaped the AML treatment paradigm. However, these advances are accompanied by questions and challenges regarding assessment of treatment efficacy over time and identification of the most accurate and clinically meaningful parameters of long‐term response. As a post‐treatment biomarker, mutation clearance has the potential to further define the quality of response to a given therapy, which can augment and refine prognosis in AML.^23^ To this end, European LeukemiaNet (ELN) has recently endorsed the achievement of MRD − CR as a key treatment goal in AML using either flow cytometry or real‐time PCR approaches, as appropriate.^7^, ^24^

In the current analysis, we evaluated treatment response and mutation clearance using a combined PCR‐ and NGS‐based approach in a subgroup of patients with *FLT3* ^mut+^ R/R AML who received gilteritinib therapy. Our findings showed that gilteritinib induced deep molecular responses and *FLT3*‐ITD clearance, as defined by a low *FLT3*‐ITD allele burden, in heavily pretreated patients with *FLT3*‐ITD+ R/R AML. Results from this analysis demonstrated a potential relationship between achievement of *FLT3*‐ITD clearance and longer OS in these patients. Patients who achieved CRc or CR/CRh with gilteritinib therapy had a higher rate of *FLT3*‐ITD clearance and longer OS than those who did not achieve CRc or CR/CRh.

A similar relationship between mutation clearance and treatment response has been observed in studies of isocitrate dehydrogenase (IDH) inhibitors in patients with *IDH1*‐ or *IDH2*‐mutated R/R AML.^25^, ^26^ In patients treated with the IDH1 inhibitor ivosidenib, the clearance of *IDH1* mutations was associated with longer OS and remission duration. Median OS was 14.5 months in patients with *IDH1* mutation clearance and 10.2 months in patients without *IDH1* mutation clearance; median duration of CR/CRh was 11.1 and 6.5 months, respectively.^25^ Following treatment with the IDH2 inhibitor enasidenib, 12 patients with *IDH2*‐mutated R/R AML had *IDH2*‐R140 mutation clearance and 10 of these patients achieved morphologic CR.^26^ Median OS was longer among patients who achieved morphologic CR with mutation clearance (22.9 months) than in those who did not achieve morphologic CR (8.8 months; *p* = 0.0153). However, among the 35 patients who achieved morphologic CR, there was no significant difference in OS between patients who had mutation clearance (*n* = 10; median OS, 22.9 months) and those who did not have mutation clearance (*n* = 25; median OS, 20.7 months).^26^

In regard to treatment response, the goal of induction therapy is to achieve morphologic CR; however, R/R patients receiving gilteritinib or other FLT3 inhibitors frequently achieve less well‐validated responses, such as CRi.^18^, ^21^ It remains to be seen whether CRi achieved after FLT3‐targeted therapy reshapes this perception. The use of CRh as a marker of response was implemented in the pivotal phase 3 ADMIRAL trial, comparing gilteritinib with salvage chemotherapy in *FLT3* ^mut+^ R/R AML,^20^ and has also been implemented in the phase 1 trial of the IDH1 inhibitor, ivosidenib, in *IDH1*‐mutated R/R AML.^25^ Given the dearth of evidence regarding the impact of CRi, CRp, or CRh on mutation clearance after FLT3 inhibitor therapy in patients with *FLT3*‐ITD+ AML, further evaluation of these responses is clearly warranted.

It is difficult to identify the specific variables that are likely to yield the best outcomes in patients treated with gilteritinib—whether a bias toward HSCT exists in patients who had *FLT3*‐ITD clearance, whether patients had *FLT3*‐ITD clearance before HSCT, or whether patients who had *FLT3*‐ITD clearance received gilteritinib maintenance therapy. However, our small sample size restricts extrapolation of our findings to the larger R/R AML population. We did not evaluate the impact of commonly occurring co‐mutations such as *NPM1*, which could potentially influence treatment response and OS. We also did not perform MFC as a means of cross‐platform validation of MRD– status in patients who achieved CR or CRc and had *FLT3*‐ITD clearance. Because results from our survival analyses did not reach statistical significance, the validity of these observations should be further tested in an appropriately powered independent validation cohort. We observed that most patients with *FLT3*‐ITD mutation clearance following gilteritinib therapy had also achieved CR with prior AML therapy and had a longer duration of response to prior treatment than patients without *FLT3*‐ITD mutation clearance. However, due to the small sample size, these observations should be interpreted with caution and further investigated in a larger population.

It is important to note that *FLT3*‐ITD mutations occur as late hits in leukemogenesis and often present in subclones.^27^, ^28^ Thus, clearance of *FLT3*‐ITD mutations is not necessarily proportional to reduction in the percentage of leukemic blasts or the clearance of clonal/pre‐leukemic cells. Recent evidence demonstrates that although gilteritinib therapy can clear *FLT3*‐ITD mutations, clones may persist in patients on the basis of karyotype or co‐mutations, suggesting that clearance of a subclone may not be indicative of MRD − status according to conventional standards.^15^ Clearance of all mutations in patients with *FLT3* ^mut+^ R/R AML who received gilteritinib therapy appears to be limited to patients who relapsed after prior HSCT.^15^ We did not assess patients with baseline point mutations in the *FLT3* tyrosine kinase domain due to the low number of patients harboring these mutations (*n* = 20) in the 120‐ and 200‐mg gilteritinib dose groups,^18^ which would have been insufficient for our analyses. Moreover, the NGS assay that was used was specifically designed for the detection of *FLT3* insertions that characterize *FLT3*‐ITD mutations. This assay allows for exquisite sensitivity as the particular *FLT3* insertion is unique for each patient, whereas a point mutation could be mistaken for noise due to inherent polymerase error. Currently, there is no available NGS assay with sufficient sensitivity (at least 1 × 10^−4^) to detect *FLT3*‐TKD point mutations.

In conclusion, this is the first study that evaluated the long‐term effect of *FLT3*‐ITD mutation clearance after treatment with a FLT3 inhibitor in patients with *FLT3* ^mut+^ R/R AML. Gilteritinib was effective in inducing *FLT3*‐ITD clearance and remission in these patients. Patients who achieved morphologic remission with *FLT3*‐ITD clearance, defined as MRD negativity, showed a trend toward longer survival than those who achieved remission with persistence of the *FLT3*‐ITD clone. Although the ELN does not currently recommend the use of *FLT3*‐ITD mutations in the analysis of MRD due to the unstable nature of these mutations,^7^ assessment of mutation clearance in patients receiving these therapies will be an important determinant of response durability in patients who achieve remission as the use of highly specific agents against FLT3 and other leukemic targets becomes more prevalent in AML.^6^, ^9^ Given that the goal of treatment is to achieve remission and prevent relapse, it will be important to determine the impact of gilteritinib therapy on mutation clearance in patients with newly diagnosed AML.

## DISCLOSURES

JK Altman reports institutional grant funding from Boehringer Ingelheim, Astellas, Agios, Celgene, Genetech, FujiFilm, and BioSight; review activities with Glycomimetrics and the US lead PI for BioSight trial; advisory board participation with Astellas, Novartis, Syros, Jannsen Pharmaceuticals, Celgene, Immune Pharmaceuticals (did not accept payment), Bristol Myers Squibb, ASH, Cancer Expert Now, Agios, Theradex, AbbVie, and Daiichi Sankyo; and personal fees from Astellas. AE Perl reports personal fees and non‐financial support from Astellas during the conduct of the study; institutional grant funding from Astellas, Bayer, BioMed Valley Discoveries, Daiichi Sankyo, Fujifilm, and Novartis; personal fees and other support from Daiichi Sankyo, Arog, Novartis, Jazz Pharmaceuticals, Takeda Oncology, AbbVie, NewLink Genetics, Asana Biosciences, and Seattle Genetics; and personal fees from Pfizer and Actinium Pharmaceuticals. JE Hill is an employee of Astellas; reports stock ownership in Ligacept, LLC; and has patents US 7862995, US 9051388, and US 9683222 issued. M Rosales and E Bahceci are employees of Astellas. MJ Levis reports grant funding from Astellas during the conduct of the study, grant funding from Novartis and FujiFilm, and personal fees from Daiichi Sankyo.

## AUTHOR CONTRIBUTIONS

JEH, EB, and MJL designed the research study; JEH performed the research; JKA, AEP, and JEH acquired the data; JEH, MR, EB, and MJL analyzed the data; JKA, AEP, JEH, MR, EB, and MJL interpreted the data; JEH drafted the manuscript; and JKA, AEP, JEH, MR, EB, and MJL critically reviewed and revised manuscript. All authors approved the final version of the manuscript.

## Supporting information

Fig S1

## Data Availability

Researchers may request access to anonymized participant‐level data, trial‐level data, and protocols from Astellas sponsored clinical trials at www.clinicalstudydatarequest.com. For the Astellas criteria on data sharing, see: https://clinicalstudydatarequest.com/Study‐Sponsors/Study‐Sponsors‐Astellas.aspx
